# Quality of Life and Related Factors in Patients With Ankylosing Spondylitis: A Cross-Sectional Study Using 36-Item Short Form Survey (SF-36)

**DOI:** 10.7759/cureus.44695

**Published:** 2023-09-05

**Authors:** Nguyen Tran Tuyet Lan, Ho Tat Bang, Tran Thi Anh Thu, Nguyen Thi My Anh, Nguyen Van Tap

**Affiliations:** 1 Department of Infection Control, Children's Hospital 1, Ho Chi Minh City, VNM; 2 Thoracic and Vascular Department, University Medical Center Ho Chi Minh City, University of Medicine and Pharmacy at Ho Chi Minh City, Ho Chi Minh City, VNM; 3 Department of Health Organization and Management, Faculty of Public Health, University of Medicine and Pharmacy at Ho Chi Minh City, Ho Chi Minh City, VNM; 4 Faculty of Public Health, University of Medicine and Pharmacy at Ho Chi Minh City, Ho Chi Minh City, VNM; 5 Department of Health Communication, Binh Thanh Medical Center, Ho Chi Minh City, VNM; 6 Faculty of Nursing and Medical Technology, University of Medicine and Pharmacy at Ho Chi Minh City, Ho Chi Minh City, VNM; 7 Faculty of Medical Management, Nguyen Tat Thanh University, Ho Chi Minh City, VNM

**Keywords:** mental health, physical health, sf-36, ankylosing spondylitis, quality of life

## Abstract

Background: Ankylosing spondylitis is a chronic condition that affects the vertebral and sacroiliac joints, causing progressive back pain and stiffness. Patients with this condition experience a lower quality of life compared to the general population, with physical health being more impacted than mental health. In Vietnam, little attention has been given to the quality of life of patients with ankylosing spondylitis, and there are conflicting studies on the factors that affect their quality of life. Therefore, it is essential to assess the quality of life of these patients to provide appropriate recommendations for improving their overall well-being.

Methodology: The descriptive cross-sectional study was conducted on patients with ankylosing spondylitis who visited University Medical Center Ho Chi Minh City from March 2022 to May 2022. Participants were interviewed face-to-face using the 36-item short form survey (SF-36) questionnaire and the visual analogue scale. In addition, some information related to ankylosing spondylitis was also collected through medical records.

Results and conclusion: Seventy-five patients met the inclusion criteria with a median age of 33 (26 - 37); men accounted for 68%. The mean physical and mental health scores on the SF-36 scale were 37.73 ± 9.30 and 47.04 ± 7.11, respectively. Young age, lower pain score and duration of illness, and higher education were associated with a high physical health score with p<0.05. Similarly, the educational level and occupation were positively correlated, and pain scores were inversely correlated with mental health with p< 0.05.

## Introduction

Ankylosing spondylitis is a chronic disease affecting the vertebral and sacroiliac joints, characterized by progressive back pain and stiff joints [1]. The disease begins at 15 to under 40 years old; men predominate over women and are closely related to the HLA-B27 antibody [2,3]. Furthermore, ankylosing spondylitis also damages peripheral joints and other organs in addition to joints, including uveitis, psoriatic dermatitis, and enteritis [4]. Naturally, chronic inflammation leading to the fusion of the vertebrae is the cause of disability and mortality in patients. Previous studies have shown that the quality of life is lower in patients with ankylosing spondylitis compared with the general population, in which the physical health is more affected than the mental health aspect [5-7]. In Vietnam, we found that the quality of life in patients with ankylosing spondylitis has received little attention. The relationships affecting the quality of life are still conflicting between studies. Therefore, it is essential to assess the quality of life in patients with ankylosing spondylitis, thereby contributing to making appropriate recommendations to improve the quality of life in patients.

The University Medical Center Ho Chi Minh City is a hospital specializing in treating various medical conditions. Among the many patients seeking treatment yearly, over 30,000 suffer from musculoskeletal diseases, including ankylosing spondylitis. However, no research has been conducted on the quality of life experienced by patients with this condition. As a result, a study was conducted to investigate the mean score of quality of life and the related factors that affect the quality of life of patients with ankylosing spondylitis at this hospital.

## Materials and methods

Objectives of the study

The objectives of this study include: (1) Determining the mean score of quality of life of ankylosing spondylitis patients visiting the Hospital of Medical University of Ho Chi Minh City using the 36-item short form survey (SF-36) scale and (2) determining the relationship between physical health (PH) score, mental health (MH) score with demographic characteristics and some clinical characteristics in ankylosing spondylitis (disease duration, age of disease onset, pain score).

Study settings and design

A cross-sectional study was conducted on patients with ankylosing spondylitis visiting University Medical Center Ho Chi Minh City from March 2022 to May 2022. The study has been approved by the Ethics Council in Biomedical Research of the University of Medicine and Pharmacy Ho Chi Minh City, No. 236/HĐĐĐ-DHYD, signed on February 28, 2022. All procedures were performed according to the Declaration of Helsinki. This work has also been reported in line with the strengthening of the reporting of cohort, cross-sectional, and case-control studies in surgery (STROCSS) criteria.

Participants, sample size, and sampling

The inclusion criteria included: (1) Patients with ankylosing spondylitis who were 18 years old or older visiting University Medical Center Ho Chi Minh City and (2) the patient consented to participate in the study. Exclusion criteria included patients with a history of autoimmune joint diseases, rheumatism, and psoriatic arthritis.

The study used a formula to calculate the sample size to estimate a mean SF-36 score of quality of life in which standard deviation ϭ = 1.35 (according to research conducted by Hai and his colleagues at Bach Mai Hospital in 2019 [8]), Z(1 -α/2) = 1.96, d =0.34 (ϭ/4). The minimum sample size was calculated to be 61. The study sample conveniently selected all patients who met the sampling criteria during the study period. The selection of patients was carried out continuously at the hospital's outpatient clinic until the minimum sample size was reached.

Data collection methods and tools

Patients were selected correctly with the inclusion and exclusion criteria, and the study's objectives were explained in detail by an investigator. After obtaining informed written consent from the patient, the interviewer conducted a face-to-face interview with a set of prepared questionnaires including demographic and socio-economic segmentations, and the characteristics of ankylosing spondylitis including the age of disease onset, duration of illness, visual analogue scale (VAS) pain scores, HLA-B27 test results and symptoms at joints and outside joints were collected through medical records. The quality of life was assessed with the SF-36 toolkit.

SF-36 toolkit

The SF-36 questionnaire is a general health survey of the Medical Outcomes Study (MOS) developed by the RAND (Research And Development) Corporation with 36 evaluation items in eight different fields and divided into two main aspects: physical health and mental health. SF-36 is currently the most widely used in over 60 countries and 120 languages. It is not only used to assess the overall quality of life, but SF-36 is also commonly used in other chronic diseases (diabetes mellitus, hypertension, chronic obstructive pulmonary disease... etc.), showing that this scale is valuable and reliable in measuring [5].

The SF-36 questionnaire is scored on a scale of 0 to 100 for each question. The higher the score, the better the quality of life and vice versa. In the same area, if any question is left blank, it is not counted, and the score for each area is calculated as the average of the sub-questions. The average of the four areas of physical activity, physical activity limit, pain, and general well-being is the physical health score, and an average of four areas of emotional well-being, perceived vitality, general well-being, and social functioning were counted as mental health scores. The overall quality of life score is calculated as the average of the two dimensions of physical health and mental health.

Statistical analysis method

Frequency and percentage were used to describe qualitative variables. Mean and standard deviation were used for a normally distributed quantitative variable and the median (interquartile range) for a variable with an abnormal distribution. The unpaired t-test measured the association between the binary variable with the PH and MH mean scores with a normal distribution. The ANOVA test was used to measure the association between the nominal and ordinal variables with the PH mean score and the MH mean score with a normal distribution. Simple linear regression was used to determine the relationship between the PH and MH mean scores and quantitative variables with normal distribution. The test was significant for p < 0.05.

## Results

During the period from March 2022 to May 2022, 75 patients were enrolled in the study. The characteristics of the participants are described in Table 1. The median age of the study was 33 (26-37), the youngest was 19 years old, and the oldest was 54. All patients were from the Kinh ethnic group. The proportion of men was two times higher than that of women, and most lived in urban areas (61.33%). More than half of those with academic degrees were from vocational school/college/university/ postgraduate. All subjects had jobs with a monthly income of over 5 million, accounting for the majority (89.33%). Nearly two-thirds of the patients were married and living with their families, making up a majority (73.33%).

**Table 1:** Demographic and socio-economic characteristics *: Mean (interquartile range)

Characteristics		Frequency	Ratio %
Age (year) *	33 (26-37)		
Gender	Male	51	68
	Female	24	32
Academic degree	Graduated from primary school	5	6.67
	Graduated from secondary school	12	16
	Graduated from high school	15	20
	Vocational school/college/university/postgraduate	43	57.33
Income (Vietnam Dong)	<5 million	8	10.67
	5 million	67	89.33
Marital status	Single	29	38.67
	Married	46	61.33
Cohabitation status	With family	55	73.33
	With friends	15	20
	Alone	5	6.67
Ethnic group	Kinh ethnic group	75	100
	Minority groups	0	0

Table 2 describes the characteristics of ankylosing spondylitis. The median age of disease onset was 26 years, the youngest was 12 years old, and the oldest was 51 years old. The disease duration was 2.5 years, the lowest was three months, and the highest was 20 years. The mean VAS score was 48.62 ± 22.79. HLA-B27 antigen was positive in up to 88% of patients. 

**Table 2:** Some characteristics of ankylosing spondylitis VAS: Visual analogue scale; HLA: human leukocyte antigen

Characteristics	Results
Age of disease onset (age)	26 (20 - 34)
Disease duration (years)	2.5 (1.25 - 4.33)
Mean VAS score (point)	48.62 ± 22.79
HLA-B27 test (positive)	88%

Symptoms are diversely distributed in specific joints in Table 3, with symptoms in the lumbar spine accounting for the highest percentage, followed by the cervical spine and resistance joints, and the lowest in the ankle joints. Extra-articular symptoms were only recorded in the eyes, with 5.33%.

**Table 3:** Features of ankylosing spondylitis

Symptoms	Frequency	Ratio %
Lumbar spine	72	96
Cervical spine	30	40
Sacroiliac joint	5	6.67
Shoulder joint	9	12
Hip joint	27	36
Knee joint	19	25.68
Ankle joint	6	8

The mean score of quality of life through the SF-36 scale was 42.38 ± 6.65 points, in which the score of physical health was lower than that of mental health. The two areas with the lowest scores were physical activity limitation and general health. Meanwhile, physical activity had the highest score, with 60 points. The specific scores of the areas are presented in Table 4.

**Table 4:** Quality of life score based on the SF-36 scale SF-36: 36-Item Short Form Survey

Aspects	Fields	Quality of Life Score	
Physical Health (PH)	Physical activity	60 (50 – 70)	37.73 ± 9.30
	Limit physical activity	25 (0 – 50)	
	Experience pain	50.20 ± 16.61	
	General health	16.67 (16.67 – 20.83)	
Mental health (MH)	Emotional limit	45.78 ± 21.06	47.04 ± 7.11
	Feel the vitality	40.73 ± 6.71	
	General mental	48 (44 – 52)	
	Social activities	53.17±13.49	
	Total score SF-36	42.38 ± 6.65	

The research showed a relationship of the physical health score with age (p = 0.008) and academic level (p = 0.016). Specifically, the older people were, the lower the PH. In contrast, people with an academic level from primary school graduation or higher had higher scores on physical health. Regarding mental health, the study also found a link between the academic level (p=0.006) and occupation (p=0.002). The higher the academic level of people, the better their mental health. Farmers had a lower mean score on mental health than the rest of the occupational groups (Table 5).

**Table 5:** Relationship between quality of life and demographic and socio-economic characteristics # Regression coefficient (95% CI) (a)  t-test with unequal variance              (b) t-test with equal variance (c) ANOVA test                                      (d) Simple linear regression PH: Physical health; MH: mental health

Characteristic	PH score	MH score
Gender		
Male	37.53 ± 8.19	47.50 ± 7.18
Female	38.15 ±11.50	46.07 ± 70
p-value	0.813(a)	0.421(b)
Age	-0.34 (-0.59 – (-0.09))^#^	-0.16 (-0.35 – 0.04)^ #^
p . value	0.008(d)	0.121(d)
Place of living		
City	37.35 ± 9.51	46.67 ± 7.61
Countryside	38.33 ± 9.08	47.63 ± 6.32
p . value	0.661(b)	0.570(b)
Academic level		
Graduated from primary school	26.83 ± 6.78	37.78 ±2.42
Graduated from secondary school	37.59 ± 8.26	44.63 ±7.36
Graduated from high school	41.97 ± 9.2	48.29 ± 60
Vocational school/College/	37.55 ± 9.01	48.35± 6.94
University/Postgraduate		
p . value	0.016(c)	0.006(c)
Occupation		
Freelance	35.46 ± 8.88	43.30 ± 6.03
Retirement	34.69 ± 5.70	50 ± 7.75
Housewife	34.58 ±15.32	38.83 ± 0.07
Office staff	37.46 ± 9.82	49.29 ± 6.63
Farmer	34.48 ± 8.40	38.67 ± 3.24
Worker	40.90 ±10.27	50.58 ± 7.04
Other	42.16 ± 8.14	49.72 ± 5.95
p . value	0.398(c)	0.002(c)
Income		
<5 million	39.40 ± 9.46	48.67 ± 7.19
5 million	37.53 ± 9.33	46.84 ± 7.13
p . value	0.594(b)	0.496(b)
Cohabitation status		
With family	38.21 ± 9.29	47.53 ± 7.07
With friends	36.96 ± 9.55	45.03 ± 7.76
Alone	34.71 ± 9.92	47.65 ± 5.51
p . value	0.683(c)	0.480(c)
Marital status		
Single	37.74 ± 9.24	45.89 ± 6.75
Married	37.72 ± 9.44	47.76 ± 7.30
p . value	0.990(b)	0.271(b)

Table 6 shows a negative correlation between the PH score with disease duration and VAS pain score. For those with an increased disease duration by one year, the mean score of PH decreased by 1.09 points. Similarly, those with pain scores increased by 1 point, and the mean PH score decreased by 0.19 points.

**Table 6:** Relationship between physical health and some characteristics of disease VAS: Visual analogue scale

Characteristics	Regression coefficient	95% CI	p-value
Age of disease onset	-0.16	(-0.40) –0.83	0.194
Duration of disease	-1.09	(-1.70) – (-0.49)	0.001
VAS pain score	-0.19	(-0.27) – (-0.10)	<0.001

The results of Table 7 show a negative correlation between mental health and VAS pain score (p=0.011). Specifically, for those who increased their pain score by 1 point, the mean MH score decreased by 0.09 points.

**Table 7:** Relationship between mental health and some characteristics of disease VAS: Visual analogue scale

Characteristics	Regression coefficient	95% CI	p-value
Age of disease onset	-0.10	(-0.29) - 0.08	0.271
Duration of disease	-0.12	(-0.62) - 0.39	0.645
VAS pain score	-0.09	(-0.16) – (-0.02)	0.011

## Discussion

The study evaluating the quality of life in patients with ankylosing spondylitis recorded the median age of the study as 33 (26-37), with male predominance and two times higher than the female rate. This result is consistent with recent studies [4,7]. Recently, the proportion of women diagnosed with ankylosing spondylitis is increasing [9-11]. The reason is that extra-articular symptoms appear more common, and pain in females differs from inflammatory pain in males [12,13].

The median age of disease onset was 26, similar to the study by Hai et al., with the mean age of onset being 25.4 ± 9.7 years [8]. The median disease duration was 2.5 years, at least three months, and at most 20 years, lower than in other studies [8,14]. The reason for this difference may come from the delay and procrastination in diagnosis [11,15]. A study of 235 patients with ankylosing spondylitis in the United States found that the mean time from symptom onset to first diagnosis took up to 8.5 years [11]. From that, it can be seen that the diagnosis of ankylosing spondylitis is still difficult due to the limited knowledge of the patient and the incorrect identification of the disease from a doctor who is not a rheumatologist because chronic back pain symptoms are very common.

Joint symptoms were highest in the lumbar and cervical spine and lowest in the sacroiliac joints. The order of symptoms in our study was similar to that of Hai's research [8]. We found that the quality of life regarding physical health was affected more than mental health. In general, this result was similar to other studies when detecting physical health had a lower score than mental health, with a cumulative mean score of 37.5 and 44.7, respectively [5]. However, the two domains of feeling vitality and general mental had lower scores than previous studies. The mental health assessment in our study was a general assessment, so the impact of post-Covid-19 on the mental health areas of the subjects in the study cannot be excluded.

The study found an association between mean PH scores and age. In addition, the relationship between the mean score of physical health and the mean score of mental health with the academic level was also recorded. However, the occupation was only related to the mental health aspect. Through simple linear regression, our study had similarities with Lucy Law's study when it showed that the longer the duration of the disease and the higher the VAS pain score, the worse the physical health [14].

The study had the strength of using a standardized and highly reliable quality-of-life assessment toolkit in many countries, including Vietnam. However, the research was still limited due to the small sample size and the lack of assessment of specific areas affecting patients' quality of life. Future studies need to determine what specific factors affect the mental health of patients with ankylosing spondylitis to provide the best healthcare support methods. Non-rheumatologists need to be extra vigilant when dealing with patients with chronic low back pain symptoms to take an approach that supports an early diagnosis or at least doesn't lead to misdiagnosis with other musculoskeletal diseases.

The research results show that the quality of life of patients with ankylosing spondylitis is still low. Medical managers and clinicians need to pay more attention to the patient's quality of life aspect to improve the quality of treatment.

## Conclusions

The study recorded the quality of life of patients with ankylosing spondylitis on average; the SF-36 mean score was 42.38 ± 6.65 points. The area of physical activity scored the highest; conversely, general health was the most affected. Young age, low VAS pain score, disease duration, and high academic level were associated with high physical health scores with p<0.05. Similarly, the educational level and occupation were positively correlated, and VAS pain scores were negatively correlated with mental health with p< 0.05.
